# Gestational systolic blood pressure trajectories and risk of adverse maternal and perinatal outcomes in Chinese women

**DOI:** 10.1186/s12884-021-03599-7

**Published:** 2021-02-22

**Authors:** Haoyue Teng, Yumei Wang, Bing Han, Jieyu Liu, Yingying Cao, Jiaxiang Wang, Xiaoyan Zhu, Jiaojiao Fu, Qi Ling, Chengqi Xiao, Zhongxiao Wan, Jieyun Yin

**Affiliations:** 1 Jiangsu Key Laboratory of Preventive and Translational Medicine for Geriatric Diseases, School of Public Health, Medical College of Soochow University, 199 Renai Road, Suzhou, 215123 China; 2 Department Of Epidemiology And Health Statistics, Medical College of Soochow University, Suzhou, China; 3 Department of Obstetrics, The First People’s Hospital of TaiCang, Suzhou, Jiangsu Province, China; 4 Department of Obstetrics and Gynecology, First Hospital of Soochow University, Suzhou, China; 5 Women and Children Health Care Center of Taicang, Suzhou, Jiangsu Province, China; 6 Suzhou Center for Disease Prevention and Control, Suzhou, 215004 Jiangsu China; 7 Department of Nutrition and Food Hygiene, School of Public Health, Soochow University, Suzhou, China

**Keywords:** Systolic blood pressure, Trajectory, Fetal outcome, Maternal outcome, Latent class growth mixture model

## Abstract

**Background:**

Associations between trajectories of systolic blood pressure (SBP) during pregnancy and pregnant outcomes remain unclear and disparate.

**Methods:**

Data of 20,353 mothers without chronic hypertension and who delivered live singletons between January, 2014 and November, 2019, was extracted from Taicang register-based cohort. Based on SBP measured during 10 to 40 weeks of gestation, SBP trajectories were explored using latent class growth mixture model, and their associations with maternal and neonatal outcomes were assessed by logistic regression analyses.

**Results:**

Six heterogeneous SBP trajectories were identified: low delayed-increasing (7.47%), low reverse-increasing (21.88%), low-stable (19.13%), medium-stable (21.64%), medium reverse-increasing (16.47%), and high stable (13.41%) trajectories. The high-stable trajectory had SBP around 125 mmHg in the 10th gestational week, and increased slightly onwards. When compared with the low-stable trajectory, the high-stable trajectory had maximally adjusted odds ratio (95% confidence interval) of 5.28 (2.76–10.10), 1.30 (1.13–1.50), 1.53 (1.12–2.08), 1.32 (1.06–1.65) and 1.64 (1.08–2.48) for gestational hypertension (GH), early-term delivery (ETD), preterm delivery (PTD), small for gestational age and low birth weight (LBW), respectively. Besides, the medium reverse-increasing trajectory showed significantly increased risk of GH and ETD, while the medium-stable trajectory had significantly elevated risk of ETD and PTD. Notably, SBP trajectories slightly but significantly improved risk discrimination of GH, ETD and LBW, over traditional risk factors.

**Conclusion:**

Women with different SBP trajectories were at varied risk of adverse maternal and fetal outcomes. Meanwhile, our study suggested that BP monitoring during pregnancy is necessary, especially for women with high SBP in early pregnancy or upward trajectory.

**Supplementary Information:**

The online version contains supplementary material available at 10.1186/s12884-021-03599-7.

## Background

Hypertensive disorders in pregnancy (HDP) include preeclampsia (PE), transient gestational hypertension, gestational hypertension (GH), white-coat hypertension, masked hypertension, chronic hypertension and chronic hypertension with superimposed PE [1]. HDP affects about 5–15% of pregnancies [2]. It is one of the leading causes of maternal morbidity and mortality [3–5], and may increase the likelihood of hypertension or other cardiovascular disease (CVD) within a decade of an affected pregnancy [6, 7]. Meanwhile, HDP are tied with adverse fetal outcomes, including low birth weight (LBW), preterm delivery (PTD), and small for gestational age (SGA) [8–10]. Children suffered such adverse birth outcomes are prone to functional disabilities, type 2 diabetes mellitus and CVD later in life [11–15]. Therefore, HDP have caught great attention in prenatal medicine and public health.

The cause and pathophysiology of HDP remain poorly understood. Blood pressure (BP) during pregnancy is highly dynamic [16, 17]. It was generally accepted that in clinically healthy pregnant women, BP falls gradually at the first trimester, reaching a nadir around 20 weeks, and then increases until delivery [18]. The absence of this mid-trimester BP drop may be an early indicator of HDP [19]. In contrast, there are also evidence suggesting that there might be no BP decline even in normal pregnant women [20, 21]. In addition, among women with such a mid-trimester BP decline, there is still substantial variation in parameters of this important turning point (such as the nadir of drop, initial timing of drop), which might also be informative for prediction of PE [22].

Gestational-age-specific reference ranges for BP in pregnancy have been proposed [23, 24], and alterations in B*P* values during pregnancy might be used as predictors for perinatal outcomes [17]. In regards to this, emerging evidence have focused on the association between BP change patterns during pregnancy and maternal, as well as perinatal outcomes. For example, a study in Hangzhou city of China reported five trajectories of systolic BP (SBP) during pregnancy. It was found that the earlier GH onset was, the higher the baseline BP was [25]. Besides, the BOSHI Study Group identified six trajectory groups for home SBP among 880 Japan pregnancies; trajectory groups with a low-steep J-curve, moderate J-curve, little high J-curve, and high J-curve were significantly associated with lower infant birth weight than the low-J-curve group [26]. Another Chinese studies conducted in Kunshan city found four distinct SBP trajectory patterns over the pregnancy period, and only pregnant women with moderate-increasing and high-decreasing BP patterns had statistically increased risk of developing LBW and PTD [27]. Considering disparities in identified BP trajectories and their associations with pregnant outcomes, further study in this field is still needed.

Traditional regression or growth curve model assume only one mean within the population, while the latent class growth mixture (LCGM) model can fit well the data of subgroups of people sharing similar development patterns [28]. Moreover, LCGM model is designed to address research questions focused on describing the trajectory, or pattern of change over time in the dependent variable, thus providing a good description of BP trends during pregnancy. SBP may be superior to diastolic BP when predicting diseases [29, 30]. What’s more, to the best of our knowledge, the added predictive potential of SBP trajectories for maternal and perinatal outcomes beyond traditional risk factors has never been examined. Therefore, we had three aims in the present study, to identify distinct SBP trajectories during pregnancy by using LCGM model; to examine the associations of different SBP trajectories with adverse maternal and perinatal outcomes; and to evaluate the clinical utility of identified SBP trajectories in predicting maternal and perinatal outcomes.

## Methods

### Study population

The study was based on a registered-based cohort study conducted in Taicang, a small but developed city in Jiangsu Province. Details of the Taicang register-based cohort was described previously [31, 32]. In brief, a total of 24,458 pregnant women who delivered live births between Jan. 1, 2014 and Nov. 30, 2019 in any hospital or community healthcare center in Taicang were enrolled. Subsequently, exclusion was successively made for 3635 individuals with fewer than three BP measurements between the 10th week and 40th gestational week, 367 subjects with polyembryony, and 103 pregnant women with chronic hypertension (≥140/90 mmHg) [31, 32]. Finally, a total of 20,353 pregnant women were included in the present study.

**Fig. 1:**
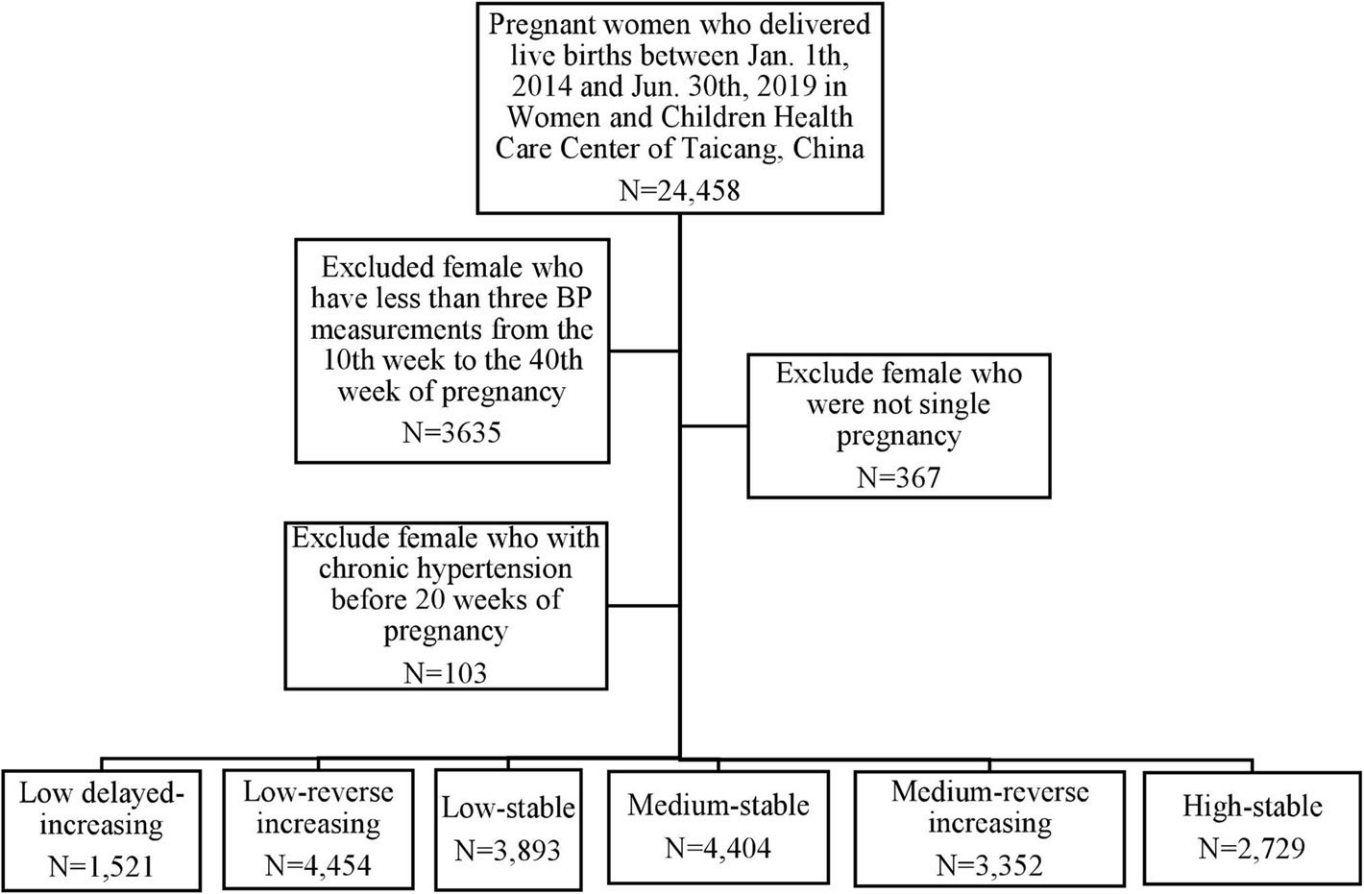
Flow chart for selection process of the study

The flowchart of the exclusion and inclusion process of our study population is presented in Fig. 1. All participants provided written informed consent. This study was approved by the ethics committee of Soochow University and Maternal and Child Healthcare Center of Taicang.

**Table 1:** Maternal and fetal characteristics at baseline according to SBP trajectories

Variables	Total population	Low-stable	Low delayed-increasing	Low reverse-increasing	Medium reverse-increasing	Medium-stable	High-stable	*P*-value
	(*n* = 20,353)	(*n* = 3893)	(*n* = 1521)	(*n* = 4454)	(*n* = 3352)	(*n* = 4404)	(*n* = 2729)	
**Maternal characteristics**								
Age (years)	27.10 ± 4.38	27.18 ± 4.29	26.80 ± 4.20	26.96 ± 4.43	27.07 ± 4.36	27.31 ± 4.43	27.48 ± 4.60	<.0001
Early pregnancy BMI (kg/m^2^)	21.61 ± 3.11	20.21 ± 2.26	20.88 ± 2.60	22.02 ± 3.11	21.03 ± 2.60	21.97 ± 3.08	23.71 ± 3.94	<.0001
Gestational age (week)	38.66 ± 1.30	38.74 ± 1.25	38.64 ± 1.25	38.59 ± 1.33	38.81 ± 1.22	38.65 ± 1.31	38.46 ± 1.44	<.0001
Primipara (n, %)	10,868 (51.89%)	869 (48.01%)	2389 (55.10%)	1892 (56.04%)	1968 (47.62%)	2139 (50.99%)	1361 (54.37%)	<.0001
Abortion in previous pregnancy (n, %)	2684 (13.19%)	219 (12.10%)	508 (11.72%)	437 (12.94%)	592 (14.32%)	568 (13.54%)	360 (14.38%)	0.0024
GDM (n, %)	4139 (20.34%)	279 (15.41%)	755 (17.41%)	739 (21.89%)	682 (16.50%)	915 (21.81%)	769 (30.72%)	<.0001
GH (n, %)	692 (3.40%)	15 (0.39%)	8 (0.53%)	57 (1.28%)	205 (6.12%)	50 (1.14%)	357 (13.08%)	<.0001
Preeclampsia/eclampsia (n, %)	182 (0.89%)	25 (0.64%)	10 (0.66%)	28 (0.63%)	30 (0.89%)	46 (1.04%)	43 (1.58%)	0.0004
Thyroid disease (n, %)	321 (1.58%)	15 (0.83%)	75 (1.73%)	48 (1.42%)	61 (1.48%)	58 (1.38%)	64 (2.56%)	0.0002
BP measurement times during pregnancy	8.24 ± 2.12	8.24 ± 2.20	8.17 ± 2.04	8.18 ± 2.07	8.41 ± 2.18	8.30 ± 2.15	8.03 ± 2.10	<.0001
SBP at first antenatal visit (mmHg)	118.13 ± 11.28	108.51 ± 7.47	104.92 ± 9.44	118.78 ± 8.85	126.42 ± 8.13	117.15 ± 7.82	129.55 ± 8.16	<.0001
**Fetal characteristics**								
Girls (n, %)	10,686 (52.50%)	921 (50.88%)	2286 (52.72%)	1728 (51.18%)	2181 (52.77%)	2264 (53.97%)	1306 (52.18%)	0.1467
Newborn weight (g)	3323.52 ± 431.85	3296.60 ± 406.76	3312.06 ± 426.98	3307.89 ± 444.97	3345.89 ± 406.46	3346.24 ± 431.01	3308.93 ± 476.67	<.0001
1-min Apgar scores	9.97 ± 0.56	9.99 ± 0.80	9.97 ± 0.35	9.98 ± 0.67	9.97 ± 0.54	9.95 ± 0.49	9.94 ± 0.63	0.0176
5-min Apgar scores	9.96 ± 0.48	9.97 ± 0.39	9.97 ± 0.39	9.96 ± 0.51	9.97 ± 0.49	9.96 ± 0.47	9.94 ± 0.64	0.1953
Low birth weight (n, %)	504 (2.48%)	28 (1.55%)	112 (2.58%)	88 (2.61%)	83 (2.01%)	90 (2.15%)	103 (4.12%)	<.0001
Small for gestational age (n, %)	1986 (9.76%)	200 (11.05%)	435 (10.03%)	374 (11.08%)	352 (8.52%)	348 (8.30%)	277 (11.07%)	<.0001
Early-term delivery (37–38) (n, %)	7420 (36.46%)	635 (35.08%)	1628 (37.55%)	1333 (39.48%)	1337 (32.35%)	1502 (35.80%)	985 (39.35%)	<.0001
Pre-term delivery (< 37) (n, %)	1033 (5.08%)	74 (4.09%)	208 (4.80%)	183 (5.42%)	160 (3.87%)	221 (5.27%)	187 (7.47%)	<.0001

*GDM* Gestational diabetes mellitus, *GH* Gestational hypertension, *BMI* Body mass index, SBP Systolic blood pressure

### Measurement of SBP

Antenatal examination for general pregnant women in the current study was based on the policy of antenatal care in Taicang city: initiated at around 12 weeks of gestation, thereafter once every 4 weeks at < 28 weeks of gestation, once every 2 weeks at 28–37 weeks of gestation, and once per week at ≥37 weeks of gestation. The measurements of BP were taken as part of routine prenatal care by physicians, and BPs values were retrieved from the computerized tracking system maintained by the study clinical institutions.

BP was measured using a calibrated mercury sphygmomanometer following a standardized protocol. In brief, all participants were seated in an upright position with back support and instructed to relax for 5 min. A cuff was placed around the nondominant upper arm, which was supported at the level of the heart, with the bladder midline over the brachial artery pulsation. We assigned an average of two sequential BPs to each record, with a minimum 2-min rest period between measurements.

### Primary outcomes and definition

The main outcome variables in our study included maternal (GH, PE/eclampsia) and fetal outcomes [LBW, SGA, PTD and early-term delivery (ETD)]. Based on the JNC7 Guideline, the 2013 American College of Obstetricians and Gynecologists (ACOG) statement defines GH as hypertension (≥140/90 mmHg) that manifested after 20 weeks’ gestation without proteinuria, PE as GH accompanied by proteinuria or other symptoms [29]. Eclampsia was defined by a patient experiencing convulsions, who had PE or severe PE, with or without albuminuria, where another cause had been ruled out [33]. PTD was defined as a live-singleton birth that occurred no later than 36 weeks of gestation, whereas ETD was defined as a live birth with a gestational age between 37 and 38 weeks [34]. SGA was defined as a gestational-age-adjusted birth weight below the tenth percentile [35]. LBW was described as an infant weight < 2500 g at delivery [36].

### Covariates

When pregnant women started their first antenatal examination, information including maternal age, early pregnancy body mass index (BMI), BP, and obstetrical history (e.g., gestation, parity, and abortion in previous pregnancy) was collected. During the following antenatal visits, status of gestational diabetes mellitus (GDM) and thyroid disease were also examined by obstetricians as a clinical routine.

GDM was considered at approximately 25 weeks of gestation when any of the following criteria were met on the 75-g oral glucose tolerance test: fasting plasma glucose level ≥ 5.1 mmol/L, ≥10 mmol/L at 1 h, and ≥ 8.5 mmol/L at 2 h [37]. Thyroid disease during pregnancy was diagnosed according to the Guidelines of the American Thyroid Association [38].

### Assessment of SBP trajectories

The number of SBP measurements achieved at < 10 weeks of gestation or at ≥40 weeks of gestation was too small to be analyzed in the current study. Therefore, we used SBP values measured between the period of 10 weeks 0 days and 40 weeks 0 days.

In our study, the change patterns of SBP were fitted by LCGM model by using Proc Traj in statistical analysis system (SAS) software 9.4. A censored normal model (CNORM) was considered appropriate due to the continuity of SBP. We mainly considered Bayesian Information Criterion (BIC) and posterior class-membership probabilities to determine the optimal trajectory model. First, the closer the BIC value is to zero, the better the model fits the data. Second, for each model involving latent trajectories, posterior class-membership probabilities were used to obtain a posterior classification of the participants in each latent trajectories to evaluate goodness-of-fit and to characterise the discrimination of latent trajectories. The higher the mean posterior class-membership probabilities within each latent trajectories, the better the model is. Third, we also retrieved the proportion of subjects classified in each latent trajectories with a posterior probability above a threshold of 0.7, indicating the proportion of subjects unambiguously classified in each latent trajectories. Proportion of subjects with high posterior probabilities(i.e. > 0.7) reaches 65%, illustrating a good classification [28]. According to the above model selection criteria, we compared 2 to 7 trajectory models, and selected six trajectories as the optimal model. The parameter estimates of each trajectory model of model with 2 to 7 trajectories are shown in Supplementary Table 1. Finally, cubic, quadratic, and linear terms were evaluated based on their statistical significance after starting with the highest polynomial. In our final model, all of the six trajectories had cubic order terms.

### Statistical analysis

Continuous and categorical variables were presented as mean ± standard deviation (SD) and frequency (percentage), respectively. Maternal and neonatal characteristics across SBP trajectories were compared by using analysis of variance (ANOVA) for normal distributed variables and Kruskal Wallis test for skewed data, respectively. We calculated odds ratio (OR) [95% Confidence level (95% Cl)] in four logistic models to evaluate the associations between SBP trajectories and adverse maternal and births outcomes. Model 1 was unadjusted. Model 2 controlled for possible influence of maternal age at delivery (in years, continuous), early pregnancy BMI (Kg/m^2^, continuous), gestation, parity, and presence of GDM. Based on Model 2, Model 3 additionally adjusted for SBP (mmHg, continuous) at the first visit for antenatal care, and SBP measurement times (continuous) during pregnancy. Model 4 further included infant sex (boys, girls) and presence of HDP (including GH, PE and eclampsia), on the basis of Model 3. We also performed sensitivity analyses to assess the robustness of our findings. What’s more, we expanded the recruited population to women with more than two times of SBP records during the antenatal examination period to avoid selection bias of the population. Eventually, the improvement in risk identification [represent as c-statistics, continuous net reclassification index (NRI), and integrated discrimination improvement (IDI)] of adding SBP trajectories over established risk model (composed by variables in Models 3 or 4) was evaluated [39, 40]. All statistical tests were performed using SAS software (version 9.4, SAS Institute, Cary, NC, USA), and differences were considered statistically significant when two-sided *P* ≤ 0.05.

## Results

### Establishment of SBP trajectory

**Fig. 2:**
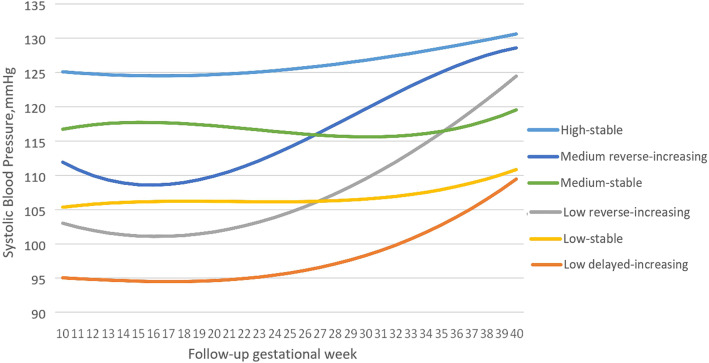
Gestational SBP trajectories from 10 to 40 gestational weeks

As shown in Figs. 2, 20,353 participants were assigned into six different subgroups: the numbers of subjects were 1521 (7.47%), 4454 (21.88%), 3893 (19.13%), 3352 (16.47%), 4404 (21.64%) and 2729 (13.41%) for the low delayed-increasing, low reverse-increasing, low-stable, medium reverse-increasing, medium-stable and high-stable patterns, respectively. Low, moderate, and high refers to SBP < 110 mmHg, 110–120 mmHg and > 120 mmHg in the 10th week of gestation, respectively. The high-stable trajectory had SBP around 125 mmHg in the 10th gestational week, and increased slightly onwards.

### Baseline characteristics

Mean maternal and gestational ages were 27.10 ± 4.38 years and 38.66 ± 1.30 weeks, respectively. Maternal and fetal demographic and baseline characteristics according to SBP trajectories are illustrated in Tables 1 and 2. In this study, the SBP measurement times during pregnancy were up to 15 (Median = 9, interquartile range = 7–10). Pregnant women belonged to the high-stable pattern presented the most adverse risk factor profile: they were older, and had the highest BMI and proportions of gestational diseases (GDM and thyroid disease). Fetuses whose mothers grouped into the high-stable were more likely to suffer ETD, PTD, LBW and SGA, and tended to have lower Apgar score at 1 min and 5 min.

**Table 2:** The associations of SBP trajectories with adverse maternal outcomes

Maternal outcomes	Low-stable	Low delayed-increasing	Low reverse-increasing	Medium reverse-increasing	Medium-stable	High-stable
	(*n* = 3893)	(*n* = 1521)	(*n* = 4454)	(*n* = 3352)	(*n* = 4404)	(*n* = 2729)
Gestational hypertension (n, %)	8 (0.53%)	57 (1.28%)	205 (6.12%)	15 (0.39%)	50 (1.14%)	357 (13.08%)
Model 1	1.00 (reference)	1.37 (0.58–3.23)	3.35 (1.90–5.93)	16.84 (9.95–28.51)	2.97 (1.67–5.30)	38.91 (23.15–65.39)
Model 2	1.00 (reference)	1.42 (0.52–3.85)	3.34 (1.72–6.48)	17.25 (9.35–31.80)	2.48 (1.25–4.93)	36.56 (19.90–67.17)
Model 3	1.00 (reference)	1.70 (0.63–4.63)	1.15 (0.58–2.26)	3.16 (1.66–6.02)	1.07 (0.53–2.14)	5.28 (2.76–10.10)
Preeclampsia/eclampsia (n, %)	10 (0.66%)	28 (0.63%)	30 (0.89%)	25 (0.64%)	46 (1.04%)	43 (1.58%)
Model 1	1.00 (reference)	1.02 (0.49–2.14)	0.98 (0.57–1.68)	1.40 (0.82–2.38)	1.63 (1.00–2.66)	2.48 (1.51–4.07)
Model 2	1.00 (reference)	1.37 (0.63–2.95)	1.07 (0.59–1.96)	1.52 (0.84–2.75)	1.72 (0.99–3.00)	2.17 (1.21–3.90)
Model 3	1.00 (reference)	1.50 (0.69–3.24)	0.81 (0.43–1.55)	0.93 (0.47–1.87)	1.36 (0.76–2.43)	1.23 (0.60–2.53)

Model 1 was unadjusted;

Model 2 was adjusted for maternal age at delivery (in years, continuous), early pregnancy BMI (Kg/m2, continuous), gestation, parity, presence of GDM;

Model 3 was additionally controlled for SBP (mmHg, continuous) at the first visit, and SBP measurement times (continuous) during pregnancy, based on model 2

### Associations of SBP trajectories with adverse maternal outcomes

As shown in Tables 2, 0.53, 1.28, 0.39, 1.14, 6.12 and 13.08% of women in the low delayed-increasing, low reverse-increasing, low-stable, medium-stable, medium reverse-increasing and high-stable patterns were defined as GH, respectively. The corresponding proportion was 0.66, 0.63, 0.64, 1.04, 0.89 and 1.58% for PE/eclampsia. Compared to women with the low-stable pattern, the medium reverse-increasing (OR = 3.16, 95% CI = 1.66–6.02) and the high-stable patterns (OR = 5.28, 95% CI = 2.76–10.10) were more likely to experience GH, after adjusting for variables in logistic regression Model 3. However, the associations between PE/eclampsia and SBP patterns were not statistically significant in any multivariate logistic regression models.

### Associations of SBP trajectories with adverse fetal outcomes

The incidences of ETD, PTD, SGA and LBW across different SBP trajectories are present in Table 3. Women demonstrated the high-stable trajectory had the highest risk of poor fetal outcomes among all the trajectories. Adjusted for variables in Model 4 and compared to women belonged to the low-stable pattern, mothers displayed the high-stable pattern had increased risk of averse birth outcomes, with OR (95% CI) of 1.30 (1.13–1.50) for ETD, 1.53 (1.12–2.08) for PTD, 1.32 (1.06–1.65) for SGA and 1.64 (1.08–2.48) for LBW, respectively. Mothers with the medium-stable trajectory also showed increased risk of ETD (OR = 1.17, 95% CI = 1.05–1.30) and PTD (OR = 1.31, 95% CI = 1.03–1.67). Meanwhile, fetuses whose mothers displayed the low reverse-increasing (OR = 1.25, 95% CI = 1.12–1.39) and the medium reverse-increasing patterns (OR = 1.27, 95% CI = 1.12–1.44) were more likely to have ETD newborns. Nevertheless, mothers with the low delayed-increasing pattern (OR = 0.57, 95% CI = 0.34–0.94) had reduced risk of LBW.

**Table 3:** The associations of SBP trajectories with adverse fetal outcomes

Fetal outcomes	Low-stable	Low delayed-increasing	Low reverse-increasing	Medium reverse-increasing	Medium-stable	High-stable
	(*n* = 3893)	(*n* = 1521)	(*n* = 4454)	(*n* = 3352)	(*n* = 4404)	(*n* = 2729)
Pre-term delivery (< 37) (n, %)	61 (4.01%)	216 (4.85%)	187 (5.58%)	150 (3.85%)	234 (5.31%)	185 (6.78%)
Model 1	1.00 (reference)	1.04 (0.77–1.41)	1.27 (1.03–1.57)	1.47 (1.18–1.84)	1.40 (1.14–1.73)	1.82 (1.46–2.26)
Model 2	1.00 (reference)	1.04 (0.76–1.42)	1.28 (1.03–1.60)	1.37 (1.08–1.72)	1.36 (1.09–1.70)	1.63 (1.28–2.07)
Model 3	1.00 (reference)	0.94 (0.68–1.31)	1.15 (0.91–1.47)	1.29 (0.97–1.71)	1.32 (1.03–1.68)	1.52 (1.12–2.07)
Model 4	1.00 (reference)	0.95 (0.68–1.32)	1.15 (0.91–1.47)	1.29 (0.98–1.72)	1.31 (1.03–1.67)	1.53 (1.12–2.08)
Early-term delivery (37–38) (n, %)	531 (34.91%)	1691 (37.97%)	1287 (38.39%)	1253 (32.19%)	1597 (36.26%)	1061 (38.88%)
Model 1	1.00 (reference)	1.13 (1.00–1.28)	1.29 (1.18–1.41)	1.31 (1.19–1.45)	1.20 (1.09–1.31)	1.34 (1.21–1.48)
Model 2	1.00 (reference)	1.14 (1.00–1.30)	1.35 (1.23–1.49)	1.37 (1.23–1.52)	1.22 (1.10–1.34)	1.39 (1.24–1.56)
Model 3	1.00 (reference)	1.11 (0.97–1.27)	1.26 (1.13–1.40)	1.26 (1.11–1.43)	1.18 (1.06–1.31)	1.26 (1.10–1.46)
Model 4	1.00 (reference)	1.12 (0.97–1.28)	1.25 (1.12–1.39)	1.27 (1.12–1.44)	1.17 (1.05–1.30)	1.30 (1.13–1.50)
Small for gestational age (n, %)	170 (11.18%)	436 (9.79%)	367 (10.95%)	351 (9.02%)	379 (8.61%)	283 (10.37%)
Model 1	1.00 (reference)	1.27 (1.05–1.54)	1.10 (0.95–1.27)	1.24 (1.06–1.45)	0.95 (0.82–1.11)	1.17 (0.99–1.38)
Model 2	1.00 (reference)	1.14 (0.93–1.41)	1.07 (0.91–1.25)	1.31 (1.11–1.55)	1.09 (0.93–1.29)	1.52 (1.27–1.82)
Model 3	1.00 (reference)	1.15 (0.93–1.42)	1.00 (0.84–1.18)	1.18 (0.96–1.44)	1.04 (0.87–1.23)	1.34 (1.08–1.68)
Model 4	1.00 (reference)	1.14 (0.93–1.41)	1.01 (0.85–1.20)	1.17 (0.96–1.43)	1.05 (0.89–1.25)	1.32 (1.06–1.65)
Low birth weight (n, %)	22 (1.45%)	119 (2.67%)	84 (2.51%)	85 (2.18%)	101 (2.29%)	93 (3.41%)
Model 1	1.00 (reference)	0.66 (0.41–1.06)	1.23 (0.93–1.63)	1.15 (0.85–1.56)	1.05 (0.79–1.41)	1.58 (1.17–2.13)
Model 2	1.00 (reference)	0.63 (0.38–1.03)	1.26 (0.94–1.69)	1.16 (0.84–1.60)	1.13 (0.83–1.54)	1.68 (1.21–2.33)
Model 3	1.00 (reference)	0.57 (0.34–0.94)	1.16 (0.84–1.59)	1.11 (0.76–1.64)	1.09 (0.78–1.52)	1.62 (1.07–2.45)
Model 4	1.00 (reference)	0.57 (0.34–0.94)	1.16 (0.84–1.59)	1.11 (0.75–1.63)	1.09 (0.78–1.51)	1.64 (1.08–2.48)

Model 1 was unadjusted;

Model 2 was adjusted for maternal age at delivery (in years, continuous), early pregnancy BMI (Kg/m2, continuous), gestation, parity, presence of GDM;

Model 3 was additionally controlled for SBP (mmHg, continuous) at the first visit, and SBP measurement times (continuous) during pregnancy, based on model 2;

Model 4 was additionally controlled for infant sex (boys, girls) and presence of hypertensive disorders in pregnancy (including GH, PE and eclampsia), on the basis of Model 3

We performed sensitivity analyses by expanding SBP records into more than twice between 10 and 40 gestational weeks. The results were similar with the analyses with at least three times of SBP records. (Supplementary Fig. 1 and Supplementary Table 2–4).

### Incremental predictive potential of SBP trajectories

Table 4 illustrates whether adding SBP trajectories to a logistic regression model consisting of other confounding factors could improve discriminative ability of individuals at poor pregnant outcomes. SBP trajectories slightly but significantly improved risk discrimination of GH, ETD and LBW, over traditional risk factors (all *P* values < 005). Specially, the incorporation of SBP trajectories to Model 3, resulted in significantly improved predictive value for GH (*c*-statistics increased from 0.835 to 0.859, *P* < 0.0001; NRI = 14.25%, *P* < 0.0001; IDI = 2.98%, *P* < 0.0001).

**Table 4:** Reclassification and Discrimination Statistics of adverse maternal and perinatal outcomes based on SBP trajectory

Clinical outcomes	Model	C statistics		Continuous NRI, %		IDI, %	
		Estimate (95% CI)	*P* value	Estimate (95% CI)	*P* value	Estimate (95% CI)	*P* value
GH	Model 3	0.835 (0.818 to 0.852)		Reference		Reference	
	Model 3+ trajectory categories	0.859 (0.844 to 0.874)	< 0.001	14.25 (0.53 to 0.68)	< 0.001	2.98 (0.002 to 0.01)	0.003
PTD	Model 4	0.833 (0.823 to 0.842)		Reference		Reference	
	Model 4+ trajectory categories	0.834 (0.824 to 0.843)	0.151	4.76 (0.09 to 0.22)	< 0.001	2.63 (0.0002 to 0.002)	0.009
ETD	Model 4	0.665 (0.657 to 0.673)		Reference		Reference	
	Model 4+ trajectory categories	0.666 (0.658 to 0.674)	0.038	2.83 (0.01 to 0.07)	< 0.001	4.36 (0.001 to 0.002)	< 0.001
SGA	Model 4	0.655 (0.642 to 0.668)		Reference		Reference	
	Model 4+ trajectory categories	0.657 (0.643 to 0.670)	0.171	3.12 (0.03 to 0.13)	0.002	2.94 (0.0002 to 0.001)	0.003
LBW	Model 4	0.665 (0.657 to 0.658)		Reference		Reference	
	Model 4+ trajectory categories	0.666 (0.658 to 0.674)	0.038	3.59 (0.08 to 0.26)	0.0003	2.77 (0.0004 to 0.002)	0.006

*NRI* net reclassification improvement, *IDI* integrated discrimination index, *CI* confidence interval, *GH* gestational hypertension, *ETD* early-term delivery, *PTD* pre-term delivery, *SGA* small for gestational age, *LBW* low birth weight

Model 3 included maternal age at delivery (in years, continuous), early pregnancy BMI (Kg/m2, continuous), gestation, parity, presence of GDM, SBP (mmHg, continuous) at the first visit, and SBP measurement times (continuous) during pregnancy;

Model 4 was additionally controlled for infant sex (boys, girls) and presence of hypertensive disorders in pregnancy (including GH, PE and eclampsia), on the basis of Model 3

## Discussion

As far as we know, there have been few studies focused on the impact of SBP trajectories on both adverse maternal and perinatal outcomes. We identified six unique SBP trajectories during pregnancy in 20,353 women without chronic hypertension in the Taicang-register based cohort. The high-stable SBP pattern with SBP > 120 mmHg in the 10th gestational week were associated with increased risk of both adverse maternal and fetal outcomes, even after adjusting for absolute SBP values. It partially supports the opinion that the newly introduced elevated BP (120–129 mmHg/ < 80 mmHg) is related with higher risk of CVD [41]. What’s more, SBP trajectories could facilitate adverse maternal and neonatal outcomes prediction, based on traditional risk factors. SBP trajectory may provide additional insight into risk of pregnant complications and allow for a low-cost office screening tool.

In our study, women within the high-stable trajectory had the highest SBP throughout gestation, and demonstrated the highest risk of GH and other neonatal outcomes. Similarly, study found that the higher the SBP during early pregnancy, the higher the risk of PE and GH [25]. Regarding the neonatal outcomes, women with a high SBP during pregnancy is more likely to delivery fetus with PTD, SGA and LBW [10, 42]. One explanation may be that high maternal BP might indicate inadequate uteroplacental perfusion, which consequently might result in intrauterine growth restriction and impaired fetal growth [10, 17, 42, 43].

The medium-stable trajectory has similar shape with the high-stable trajectory, and had increased probability of having PTD infants. In our study, we also found that pregnant women demonstrated the low reverse-increasing and the medium reverse-increasing trajectories have increased risk of GH or ETD. In line with our results, studies showed that women with an upward SBP trajectory have an increased risk of pregnant complications, compared with downward [44] or low-stable SBP trajectories [26, 27].

The current study also suggested that association magnitudes between SBP trajectories and the above-mentioned neonate outcomes depended mainly on the absolute SBP values in the third trimester. Consistently, studies found that BP elevation from the second trimester to the third trimester was associated with an increased risk of adverse birth outcomes [10, 45]. What’s more, a Chinese study found a dose-response relationship between maternal BP and adverse birth outcomes, and BP in the third trimester showed the strongest associations [46].

Based on our data, upward trajectories of SBP increased the risk of GH or fetal complications. The pregnancies’ risk of maternal and neonatal complications may substantially change beyond her initial SBP values. Our findings indicate that not only BP at an initial prenatal visit concerns, but BP elevation during pregnancy should also be the cardinal aspects of optimal antenatal care. Meanwhile, we are the first to explore the clinical utility of BP trajectory for predicting poor maternal and neonatal outcomes. As a simple, noninvasive and cost-effective method, SBP trajectories may facilitate the discrimination of women at high risk of poor outcomes, especially for GH.

The main advantage of the current study is the relatively large sample size, which could provide sufficient capacity to estimate the association between SBP trajectories and risk of adverse pregnant outcomes. Besides, community-based study design and data extracted from computerized tracking systems, may contribute to robust and reliable results through reducing selection and recall bias and contri. Nevertheless, there were also some limitations that should be concerned. First, our analysis was based on a pregnant population recruited in Taicang city of Jiangsu Province, China, which may not represent the feature of other regions. Further exploration conducted in other population are still needed. Second, because of the low frequency of BP measurements beyond 10–40 gestational weeks in the Taicang registered-based cohort, our study only focused on SBP trajectories from the 10th to the 40th gestational weeks. Third, trombophilia is one of the major etiological factor of HDP, intrauterine growth restriction as well as neonatal small birth weight. However, the change of maternal coagulation status during pregnancy, which may be an very important mediating factor for the association between adverse pregnancy outcomes and SBP trajectories, were unluckily unavailable in the current study. Further studies are encouraged to shed light in this field. Fourth, diet and lifestyle of pregnant women were unavailable in the current study; thus, we were unable to control the influence of these confounding.

## Conclusions

The current study identified six SBP trajectories during pregnancy in a relatively large sample of Chinese pregnant women. It was found that women in the high-stable pattern had increased risk of both adverse maternal and fetal outcomes. Additionally, SBP trajectories could help in prediction of GH, ETD, and SGA. Further study evaluating the associations between BP trajectory and other pregnant or perinatal outcomes are warranted.

## Supplementary Information

**Additional file 1: Supplementary Fig. 1.** Gestational SBP trajectories from 10 to 40 gestational weeks if participants with ≥2 records were included.

**Additional file 2: Supplementary Table 1.** Latent Class Growth models results. **Supplementary Table 2.** The associatio Mixture ns of SBP trajectories with adverse maternal outcomes. **Supplementary Table 3.** The associations of SBP trajectories with adverse fetal outcomes. **Supplementary Table 4.** Reclassification and Discrimination Statistics of adverse maternal and perinatal outcomes based on SBP trajectory.

## Data Availability

The datasets used and/or analyzed during the current study are available from the corresponding author on reasonable request.

## Abbreviations

HDP: Hypertensive disorders of pregnancy
GH: Gestational hypertension
SBP: Systolic blood pressure
DBP: Diastolic blood pressure
PE: preeclampsia
PTD: preterm delivery
SGA: small for gestational age
LBW: Low birth weight
BMI: Body mass index
OR: Odds ratio
95% CI: 95% confidence interval
GDM: Gestational diabetes mellitus
