# Supplementary material for: Gestational systolic blood pressure trajectories and risk of adverse maternal and perinatal outcomes in Chinese women
Source: BMC Pregnancy Childbirth. 2021 Feb 22;21:155. doi: 10.1186/s12884-021-03599-7 (PMC7898428; doi:10.1186/s12884-021-03599-7)
Supplement: Supplementary file 1 — Additional file 1: Supplementary Fig. 1. Gestational SBP trajectories from 10 to 40 gestational weeks if participants with ≥2 records were included. [file 12884_2021_3599_MOESM1_ESM.docx]

**Supplementary Figure 1.** Gestational SBP trajectories from 10 to 40 gestational weeks if participants with ≥2 records were included.
