# Supplementary material for: Gestational systolic blood pressure trajectories and risk of adverse maternal and perinatal outcomes in Chinese women
Source: BMC Pregnancy Childbirth. 2021 Feb 22;21:155. doi: 10.1186/s12884-021-03599-7 (PMC7898428; doi:10.1186/s12884-021-03599-7)
Supplement: Supplementary file 2 — Additional file 2: Supplementary Table 1. Latent Class Growth models results. Supplementary Table 2. The associatio Mixture ns of SBP trajectories with adverse maternal outcomes. Supplementary Table 3. The associations of SBP trajectories with adverse fetal outcomes. Supplementary Table 4. Reclassification and Discrimination Statistics of adverse maternal and perinatal outcomes based on SBP trajectory. [file 12884_2021_3599_MOESM2_ESM.docx]

**Supplementary Table 1.** Latent Class Growth Mixture models results.

| Nb. Latent classes | Polynomial  degree | Log-Likelihood | BIC | % Participants per class | Mean posterior probabilities | Posterior probabilities > 0.7 (%) |
| --- | --- | --- | --- | --- | --- | --- |
| 2 | Cubic | -610271.8 | −610320.8 | 52.68/47.32 | 0.95/0.95 | 94.22/93.45 |
| 3 | Cubic | -603874.5 | −603948.6 | 27.02/48.25/24.74 | 0.91/0.90/0.92 | 89.02/88.12/89.68 |
| 4 | Cubic | -601561.6 | −601660.4 | 26.93/26.90/22.72/23.44 | 0.91/0.80/0.79/0.91 | 87.31/70.60/68.22/88.35 |
| 5 | Cubic | -599577.5 | −599701.0 | 14.31/30.28/18.43/21.59/15.40 | 0.88/0.82/0.79/0.79/0.90 | 83.65/74.72/67.13/67.70/85.23 |
| 6 | Cubic | -598090.9 | −598239.2 | 7.47/21.88/16.47/19.13/13.41/21.64 | 0.87/0.77/0.78/0.78/0.88/0.79 | 80.68/65.16/66.30/65.70/84.94/67.71 |
| 7 | Cubic | -597391.0 | −597564.0 | 5.52/19.42/17.50/16.11/20.00/8.93/12.49 | 0.86/0.76/0.79/0.77/0.75/0.86/0.75 | 80.92/62.37/68.31/65.75/61.09/80.40/60.00 |

Reported are: the number of latent class considered, the polynomial form of the model, the maximum Log-Likelihood , the Bayesian information Criterion (BIC), and for models with 2 or more classes, the a-posteriori classification of subjects in each class (%), the mean of posterior probabilities in each latent class, and the % of subjects classified in each class with a posterior probability above 0.7.

| \| **Supplementary Table 2.** The associations of SBP trajectories with adverse maternal outcomes \| \| \| \| \| \| \| \| --- \| --- \| --- \| --- \| --- \| --- \| --- \| \| Maternal outcomes \| Low-stable \| Low delayed-increasing \| Low reverse-increasing \| Medium reverse-increasing \| Medium-stable \| High-stable \| \| Trajectory 4  (n=3893) \| Trajectory 1  (n=1521) \| Trajectory 2  (n=4454) \| Trajectory 3  (n=3352) \| Trajectory 5  (n=4404) \| Trajectory 6  (n=2729) \| \| Gestational hypertension (n, %) \| 8 (0.53%) \| 57 (1.28%) \| 205 (6.12%) \| 15 (0.39%) \| 50 (1.14%) \| 357 (13.08%) \| \| Model 1 \| 1.00 (reference) \| 1.37 (0.58-3.23) \| 3.35 (1.90-5.93) \| 16.84 (9.95-28.51) \| 2.97 (1.67-5.30) \| 38.91 (23.15-65.39) \| \| Model 2 \| 1.00 (reference) \| 1.42 (0.52-3.85) \| 3.34 (1.72-6.48) \| 17.25 (9.35-31.80) \| 2.48 (1.25-4.93) \| 36.56 (19.90-67.17) \| \| Model 3 \| 1.00 (reference) \| 1.70 (0.63-4.63) \| 1.15 (0.58-2.26) \| 3.16 (1.66-6.02) \| 1.07 (0.53-2.14) \| 5.28 (2.76-10.10) \| \| Preeclampsia/eclampsia (n, %) \| 10 (0.66%) \| 28 (0.63%) \| 30 (0.89%) \| 25 (0.64%) \| 46 (1.04%) \| 43 (1.58%) \| \| Model 1 \| 1.00 (reference) \| 1.02 (0.49-2.14) \| 0.98 (0.57-1.68) \| 1.40 (0.82-2.38) \| 1.63 (1.00-2.66) \| 2.48 (1.51-4.07) \| \| Model 2 \| 1.00 (reference) \| 1.37 (0.63-2.95) \| 1.07 (0.59-1.96) \| 1.52 (0.84-2.75) \| 1.72 (0.99-3.00) \| 2.17 (1.21-3.90) \| \| Model 3 \| 1.00 (reference) \| 1.50 (0.69-3.24) \| 0.81 (0.43-1.55) \| 0.93 (0.47-1.87) \| 1.36 (0.76-2.43) \| 1.23 (0.60-2.53) \| \| Model 1 was unadjusted; \| \| \| \| \| \| \| \| Model 2 was adjusted for maternal age at delivery (in years, continuous), early pregnancy BMI (Kg/m2, continuous), gestation, parity, presence of GDM; \| \| \| \| \| \| \| \| Model 3 was additionally controlled for SBP (mmHg, continuous) at the first visit, and SBP measurement times (continuous) during pregnancy, based on model 2. \| \| \| \| \| \| \| |
| --- | --- | --- | --- | --- | --- | --- | --- | --- | --- | --- | --- | --- | --- | --- | --- | --- | --- | --- | --- | --- | --- | --- | --- | --- | --- | --- | --- | --- | --- | --- | --- | --- | --- | --- | --- | --- | --- | --- | --- | --- | --- | --- | --- | --- | --- | --- | --- | --- | --- | --- | --- | --- | --- | --- | --- | --- | --- | --- | --- | --- | --- | --- | --- | --- | --- | --- | --- | --- | --- | --- | --- | --- | --- | --- | --- | --- | --- | --- | --- | --- | --- | --- | --- | --- | --- | --- | --- | --- | --- | --- | --- | --- | --- | --- | --- | --- | --- |

| **Supplementary Table 3. The associations of SBP trajectories with adverse fetal outcomes.** | | | | | | |
| --- | --- | --- | --- | --- | --- | --- |
| Fetal outcomes | Low-stable | Low delayed-increasing | Low reverse-increasing | Medium reverse-increasing | Medium-stable | High-stable |
|  | Trajectory 4  (n=3893) | Trajectory 1  (n=1521) | Trajectory 2  (n=4454) | Trajectory 3  (n=3352) | Trajectory 5  (n=4404) | Trajectory 6  (n=2729) |
| Pre-term delivery (<37) (n, %) | 61 (4.01%) | 216 (4.85%) | 187 (5.58%) | 150 (3.85%) | 234 (5.31%) | 185 (6.78%) |
| Model 1 | 1.00 (reference) | 1.04 (0.77-1.41) | 1.27 (1.03-1.57) | 1.47 (1.18-1.84) | 1.40 (1.14-1.73) | 1.82 (1.46-2.26) |
| Model 2 | 1.00 (reference) | 1.04 (0.76-1.42) | 1.28 (1.03-1.60) | 1.37 (1.08-1.72) | 1.36 (1.09-1.70) | 1.63 (1.28-2.07) |
| Model 3 | 1.00 (reference) | 0.94 (0.68-1.31) | 1.15 (0.91-1.47) | 1.29 (0.97-1.71) | 1.32 (1.03-1.68) | 1.52 (1.12-2.07) |
| Model 4 | 1.00 (reference) | 0.95 (0.68-1.32) | 1.15 (0.91-1.47) | 1.29 (0.98-1.72) | 1.31 (1.03-1.67) | 1.53 (1.12-2.08) |
| Early-term delivery (37-38) (n, %) | 531 (34.91%) | 1691 (37.97%) | 1287 (38.39%) | 1253 (32.19%) | 1597 (36.26%) | 1061 (38.88%) |
| Model 1 | 1.00 (reference) | 1.13 (1.00-1.28) | 1.29 (1.18-1.41) | 1.31 (1.19-1.45) | 1.20 (1.09-1.31) | 1.34 (1.21-1.48) |
| Model 2 | 1.00 (reference) | 1.14 (1.00-1.30) | 1.35 (1.23-1.49) | 1.37 (1.23-1.52) | 1.22 (1.10-1.34) | 1.39 (1.24-1.56) |
| Model 3 | 1.00 (reference) | 1.11 (0.97-1.27) | 1.26 (1.13-1.40) | 1.26 (1.11-1.43) | 1.18 (1.06-1.31) | 1.26 (1.10-1.46) |
| Model 4 | 1.00 (reference) | 1.12 (0.97-1.28) | 1.25 (1.12-1.39) | 1.27 (1.12-1.44) | 1.17 (1.05-1.30) | 1.30 (1.13-1.50) |
| Small for gestational age (n, %) | 170 (11.18%) | 436 (9.79%) | 367 (10.95%) | 351 (9.02%) | 379 (8.61%) | 283 (10.37%) |
| Model 1 | 1.00 (reference) | 1.27 (1.05-1.54) | 1.10 (0.95-1.27) | 1.24 (1.06-1.45) | 0.95 (0.82-1.11) | 1.17 (0.99-1.38) |
| Model 2 | 1.00 (reference) | 1.14 (0.93-1.41) | 1.07 (0.91-1.25) | 1.31 (1.11-1.55) | 1.09 (0.93-1.29) | 1.52 (1.27-1.82) |
| Model 3 | 1.00 (reference) | 1.15 (0.93-1.42) | 1.00 (0.84-1.18) | 1.18 (0.96-1.44) | 1.04 (0.87-1.23) | 1.34 (1.08-1.68) |
| Model 4 | 1.00 (reference) | 1.14 (0.93-1.41) | 1.01 (0.85-1.20) | 1.17 (0.96-1.43) | 1.05 (0.89-1.25) | 1.32 (1.06-1.65) |
| Low birth weight (n, %) | 22 (1.45%) | 119 (2.67%) | 84 (2.51%) | 85 (2.18%) | 101 (2.29%) | 93 (3.41%) |
| Model 1 | 1.00 (reference) | 0.66 (0.41-1.06) | 1.23 (0.93-1.63) | 1.15 (0.85-1.56) | 1.05 (0.79-1.41) | 1.58 (1.17-2.13) |
| Model 2 | 1.00 (reference) | 0.63 (0.38-1.03) | 1.26 (0.94-1.69) | 1.16 (0.84-1.60) | 1.13 (0.83-1.54) | 1.68 (1.21-2.33) |
| Model 3 | 1.00 (reference) | 0.57 (0.34-0.94) | 1.16 (0.84-1.59) | 1.11 (0.76-1.64) | 1.09 (0.78-1.52) | 1.62 (1.07-2.45) |
| Model 4 | 1.00 (reference) | 0.57 (0.34-0.94) | 1.16 (0.84-1.59) | 1.11 (0.75-1.63) | 1.09 (0.78-1.51) | 1.64 (1.08-2.48) |
| Model 1 was unadjusted; | | | | | | |
| Model 2 was adjusted for maternal age at delivery (in years, continuous), early pregnancy BMI (Kg/m2, continuous), gestation, parity, presence of GDM; | | | | | | |
| Model 3 was additionally controlled for SBP (mmHg, continuous) at the first visit, and SBP measurement times (continuous) during pregnancy, based on model 2;  Model 4 was additionally controlled for infant sex(boys, girls) and presence of hypertensive disorders in pregnancy (including GH, PE and eclampsia), on the basis of Model 3. | | | | | | |

| \| **Supplementary Table 4.** Reclassification and Discrimination Statistics of adverse maternal and perinatal outcomes based on SBP trajectory \| \| \| \| \| \| \| \| \| \| \| --- \| --- \| --- \| --- \| --- \| --- \| --- \| --- \| --- \| --- \| \| Clinical outcomes \| Model \| C statistics \| \|  \| Continuous NRI, % \| \|  \| IDI, % \| \| \| Estimate (95% CI) \| *P* value \|  \| Estimate (95% CI) \| *P* value \| Estimate (95% CI) \| *P* value \| \| GH \| Model 3 \| 0.835 (0.818 to 0.852) \|  \|  \| Reference \|  \|  \| Reference \|  \| \| Model 3+ trajectory categories \| 0.859 (0.844 to 0.874) \| <0.001 \|  \| 14.25 (0.53 to 0.68) \| <0.001 \|  \| 2.98 (0.002 to 0.01) \| 0.003 \| \| PTD \| Model 4 \| 0.833 (0.823 to 0.842) \|  \|  \| Reference \|  \|  \| Reference \|  \| \| Model 4+ trajectory categories \| 0.834 (0.824 to 0.843) \| 0.151 \|  \| 4.76 (0.09 to 0.22) \| <0.001 \|  \| 2.63 (0.0002 to 0.002) \| 0.009 \| \| ETD \| Model 4 \| 0.665 (0.657 to 0.673) \|  \|  \| Reference \|  \|  \| Reference \|  \| \| Model 4+ trajectory categories \| 0.666 (0.658 to 0.674) \| 0.038 \|  \| 2.83 (0.01 to 0.07) \| <0.001 \|  \| 4.36 (0.001 to 0.002) \| <0.001 \| \| SGA \| Model 4 \| 0.655 (0.642 to 0.668) \|  \|  \| Reference \|  \|  \| Reference \|  \| \| Model 4+ trajectory categories \| 0.657 (0.643 to 0.670) \| 0.171 \|  \| 3.12 (0.03 to 0.13) \| 0.002 \|  \| 2.94 (0.0002 to 0.001) \| 0.003 \| \| LBW \| Model 4 \| 0.665 (0.657 to 0.658) \|  \|  \| Reference \|  \|  \| Reference \|  \| \| Model 4+ trajectory categories \| 0.666 (0.658 to 0.674) \| 0.038 \|  \| 3.59 (0.08 to 0.26) \| 0.0003 \|  \| 2.77 (0.0004 to 0.002) \| 0.006 \| \| NRI, net reclassification improvement; IDI, integrated discrimination index; CI, confidence interval; GH, gestational hypertension; ETD, early-term delivery; PTD, pre-term delivery; SGA, small for gestational age; LBW, low birth weight. \| \| \| \| \| \| \| \| \| \| \| Model 3 included maternal age at delivery (in years, continuous), early pregnancy BMI (Kg/m2, continuous), gestation, parity, presence of GDM, SBP (mmHg, continuous) at the first visit, and SBP measurement times (continuous) during pregnancy;  Model 4 was additionally controlled for infant sex(boys, girls) and presence of hypertensive disorders in pregnancy (including GH, PE and eclampsia), on the basis of Model 3. \| \| \| \| \| \| \| \| \| \| |
| --- | --- | --- | --- | --- | --- | --- | --- | --- | --- | --- | --- | --- | --- | --- | --- | --- | --- | --- | --- | --- | --- | --- | --- | --- | --- | --- | --- | --- | --- | --- | --- | --- | --- | --- | --- | --- | --- | --- | --- | --- | --- | --- | --- | --- | --- | --- | --- | --- | --- | --- | --- | --- | --- | --- | --- | --- | --- | --- | --- | --- | --- | --- | --- | --- | --- | --- | --- | --- | --- | --- | --- | --- | --- | --- | --- | --- | --- | --- | --- | --- | --- | --- | --- | --- | --- | --- | --- | --- | --- | --- | --- | --- | --- | --- | --- | --- | --- | --- | --- | --- | --- | --- | --- | --- | --- | --- | --- | --- | --- | --- | --- | --- | --- | --- | --- | --- | --- | --- | --- | --- | --- | --- | --- | --- | --- | --- | --- | --- | --- | --- | --- | --- | --- | --- | --- | --- | --- | --- | --- | --- | --- | --- |
